# Meaning in life as a protective factor against depression

**DOI:** 10.3389/fpsyg.2023.1180082

**Published:** 2023-07-17

**Authors:** Marina Baquero-Tomás, Mª Dolores Grau, Adoración-Reyes Moliner, Alejandro Sanchis-Sanchis

**Affiliations:** ^1^Escuela de Doctorado, Universidad Católica de Valencia San Vicente Mártir, Valencia, Spain; ^2^Facultad de Psicología de la Universidad Católica de Valencia San Vicente Mártir, Valencia, Spain; ^3^Campus Capacitas-UCV, Valencia, Spain

**Keywords:** meaning in life, emotional regulation, emotional dysregulation, depression, college students

## Abstract

The main objective of this study was to analyze the mediating role that meaning in life has between emotion dysregulation and depressive symptomatology in Spanish university students. Five hundred and sixty-six Spanish university students participated in the study. All of them completed the Difficulties in Emotion Regulation Scale (DERS), the Patient Health Questionnaire (PHQ-9) and the Purpose in Life Test (PIL-10). A mediation model was performed to test the hypothesis that meaning in life mediates the effect of emotional dysregulation on depressive symptomatology in college students. The results show a positive, moderate, and statistically significant correlation between depression and emotional dysregulation. In addition, a negative, moderate, and statistically significant correlation was found between depression and meaning in life, and a negative, small, and statistically significant correlation between emotional dysregulation and meaning in life. Finally, the results of the mediation model evidence the role of meaning in life in different emotional dysregulation strategies and depressive symptomatology. These findings suggest the importance of incorporating the meaning in life variable in the development and implementation of prevention and treatment programs for psychological disorders.

## Introduction

Depression is one of the most prevalent psychological disorders (James et al., 2018). It is estimated that the prevalence in Spain is of 4.13% (Global Health Data Exchange, 2017), and the annual incidence rate is of over 7–8 cases/1000 persons/year (Vieta et al., 2021). Several studies warn of the high prevalence and the increase of depressive symptomatology in university students (Auerbach et al., 2016; Li et al., 2022; Liu et al., 2022). For example, a recent systematic review and meta-analysis reports a prevalence of depressive symptoms in university students over 27% (Fernandes et al., 2023).

Depressive disorders are characterized by the presence of a persistent feeling of sadness, irritability, or emptiness, along with a notable decrease in energy that significantly impacts various aspects of individuals’ lives (American Psychiatric Association, 2013). An important concept to understand depressive symptomatology is emotion regulation (ER). ER refers to the “processes by which we influence which emotions we have, when we have them, and how we experience and express these emotions” (Gross, 1999, p. 557) In the last decade, further research has argued that difficulties in ER are central to the development and maintenance of psychopathology (Aldao and Dixon-Gordon, 2014; Gratz et al., 2015; Sheppes et al., 2015).

Individuals with depressive symptoms often experience difficulties in identifying, understanding, and expressing their emotions, as well as managing unpleasant emotions. This can lead them to excessively control their unpleasant emotions, which paradoxically can result in an increase of them (Barlow et al., 2011a,b). In fact, ER processes have been widely recognized as a transdiagnostic factor in numerous psychological disorders (Aldao, 2012; Krueger and Eaton, 2015; Norton and Paulus, 2015; Fernández et al., 2016; Sloan et al., 2017; Cludius et al., 2020) such as depression, anxiety, substance abuse, eating disorders (see Aldao et al., 2010 for a meta-analytic review) and borderline personality disorder (Sorgi-Wilson and McCloskey, 2022). Certain ER strategies are considered adaptive, as they have been associated with lower levels of psychopathology. Conversely, other strategies, considered maladaptive ER strategies, have been linked to the onset and maintenance of various psychological disorders, being depressive disorders more relevant (Schäfer et al., 2017; Van Beveren et al., 2018; Reyes-Martín et al., 2022).

Similar to the use of maladaptive ER strategies, deficits in emotional functioning have been associated with elevated levels of symptoms in numerous psychological disorders, including depression (Weiss et al., 2013; Wong et al., 2013; Buckholdt et al., 2014; Stepp et al., 2014). In this regard, empirical studies indicate that general ER abilities, including emotional clarity, conscientiousness, and tolerance, are negatively associated with the development of psychopathology (Saarijärvi et al., 2001) and with the use of maladaptive ER strategies (Jeffries et al., 2016). Scientific literature indicates that individuals prone to depression often rely on maladaptive ER strategies and struggle with implementing adaptive or effective ER strategies. They frequently experience difficulties in emotional awareness, exhibit reduced clarity, and have lower emotional tolerance (Joormann and Stanton, 2016; Liu and Thompson, 2017). These deficits in emotional awareness and clarity can impede the identification of emotions and hinder effective ER (Gross, 2015). Overall, difficulties in ER appears to be a contributing factor in both the onset and recurrence of depressive episodes (Visted et al., 2018).

An alternative framework in the study of ER and psychopathology examines broader deficits in functioning and ER (Mennin and Farach, 2007; Bradley et al., 2011). Gratz and Roemer (2004) proposed a prominent model that defines ER as a multidimensional construct with four key aspects: (a) awareness, understanding, and acceptance of emotional experiences; (b) the ability to engage in goal-directed behaviors and inhibit impulsive behaviors during negative emotions; (c) flexible use of situationally appropriate strategies to modulate emotional intensity and duration; and (d) willingness to experience negative emotions while pursuing meaningful life activities. According to this model, deficits in any of these domains indicate emotional dysregulation. The Difficulties in Emotion Regulation Scale (DERS; Gratz and Roemer, 2004) was developed to measure this multidimensional conceptualization of ER.

On the other hand, meaning in life (MIL) is another concept of considerable relevance that has been incorporated into the literature as a variable closely related to depressive symptomatology. MIL may prove to be a protective factor against depression by acting as a buffer against negative events (Wong, 2014; Arslan et al., 2021), promoting reappraisal and coping strategies, decreasing ruminative thinking style (Schäefer et al., 2013), and thereby facilitating more adaptive coping (Hooker et al., 2018; Eisenbeck et al., 2021; Wong et al., 2021; Ward et al., 2023). Frankl (1965, 2006) defined MIL as the fundamental motivational force of human beings and a prerequisite for personal self-fulfillment. Wong (1989) conceptualized MIL as an individual cognitive system that imparts personal significance to life. In this context, MIL encompasses the coherence of one’s own life, a sense of fulfillment and purpose in living, and an authentic and meaningful existence (Przepiorka, 2012). It has also been described as a multidimensional construct consisting of three key dimensions: coherence, purpose, and significance (Martela and Steger, 2016). Thus, MIL can be understood as a network of connections, interpretations, values, and evaluations that render our experiences comprehensible, guide our efforts towards a desired future, and provide a sense that our life matters and is worth living (Marco et al., 2020a).

MIL is positively associated with perception and experience of freedom, responsibility and self-determination, fulfilment of life goals, positive view of life, of the future and of oneself, and self-realization (Wong, 2011; Przepiorka, 2012; Martela and Steger, 2016). From this perspective, it is theorised that when the MIL is not achieved, frustration originates, which would be associated with hopelessness characterized by doubt about the MIL that would manifest itself in a state of boredom, perception of lack of control over one’s own life and absence of vital goals (Frankl, 1985; García-Alandete et al., 2009). In this sense, low levels of MIL would be associated with psychopathology (Yalom, 1980; Marco et al., 2017a,b, 2020a,b; García-Alandete et al., 2018) while achievement in MIL would be related to personal dedication, the presence of defined values and life goals, and life satisfaction and psychological well-being (García-Alandete, 2015). Many studies show that having higher levels of MIL is associated with better health and psychological functioning (e.g., Haugan, 2014; Czekierda et al., 2017; Miao and Gan, 2018).

According to Park (2013), MIL plays an essential role in an individual’s ability to cope with stressful situations, affecting both coping strategy choice and adjustment after managing. Several studies have highlighted that individuals with higher levels of MIL have adequate emotion regulation (Marco et al., 2017a) and more emotion-focused coping (Ward et al., 2023). In addition, cumulating evidence has documented the association of MIL with positive affect (Miao et al., 2016; Hooker et al., 2018), and thus, psychological well-being (Yalçın and Malkoç, 2015; Krok, 2017). In this regard, Steger (2009) considered positive affect as a side effect of MIL. In a meta-analysis, Pinquart (2002) highlighted that the association between MIL and depression was more robust in young adults than in older adults. This finding is congruent with literature pointing to how low MIL is associated with depression (Marco et al., 2020a; Sun et al., 2022).

Furthermore, Marco et al. (2021), proposed the mediating role of MIL between emotional dysregulation and variables related to the psychopathology of eating disorders such as depression. On the other hand, Marco et al. (2020a) include MIL as a mediator of cognitive behavioral intervention for depression. This is interesting for the main objective of this study, which aims to analyze the relationship between emotional dysregulation and depression and the mediating role that MIL has between difficulties in ER and depressive symptomatology in Spanish college students.

## Materials and methods

### Participants

A total of 566 participants, all of whom were Spanish university students living in Spain, took part in this study. The sample consisted of 137 males (24.2%) and 429 females (75.8%), with a mean age of 21.98 years (SD = 4.83). The distribution of participants across academic years was as follows: 31.3% first-year undergraduate, 14.4% second-year undergraduate, 24.5% third-year undergraduate, 14.4% fourth-year undergraduate, 1.6% fifth-year undergraduate, 0.2% sixth-year undergraduate, and 13.6% postgraduate. For the inclusion criteria, participants were required to be university students enrolled in a Spanish university and fluent in Spanish. The exclusion criteria involve participants who report any pre-existing diseases, disorders, or neurological conditions that could potentially influence the outcomes of the study, as carried out in previous literature (Murphy et al., 2021; Bresó-Grancha et al., 2022).

### Instruments

The following instruments were used for this study:

*A sociodemographic questionnaire* elaborated for the present study includes questions about age, sex, academic year, degree, city and center where the participants were studying at university.

*Difficulties in Emotion Regulation Scale* (DERS; Gratz and Roemer, 2004; Hervás and Jódar, 2008). This scale evaluates difficulties in ER in adults. It is a self-report questionnaire with a 5-point Likert-type response. The original version consists of 36 items divided into six factors: Conscientiousness (lack of awareness of emotions), Clarity (lack of emotional clarity), Impulse (difficulties in controlling impulsive behaviors when distressed), Goals (difficulties in goal involvement), Non-acceptance (non-acceptance of negative emotional responses) and Strategies (limited access to effective emotional regulation strategies). In this study we used the Spanish adapted version. The main difference with the original scale is that the Spanish version contains 28 items distributed in 5 subscales. Specifically, the items referring to: “Difficulties in impulse control” and “Limited access to regulation strategies,” were part of a single factor named “Lack of control.” The remaining factors are: Everyday Interference, Emotional Inattention, Emotional Confusion and Non-Acceptance. Higher scores indicate more difficulties in ER. The psychometric properties of this instrument are adequate both in the original version and in the Spanish adaptation, with good internal consistency (*α* = 0.93) and test–retest reliability (*r* = 0.88). Regarding the data of this study, excellent internal consistency was found for the total scale, *α* = 0.94. and the subscales, Inattention (*α* = 0.81), Confusion (α = 0.84), Non-Acceptance (*α* = 0.90), Interference (*α* = 0.89), and Lack of control (*α* = 0.91).

*Patient Health Questionnaire* (PHQ-9; Diez-Quevedo et al., 2001; Kroenke et al., 2001). This is a screening test for assessment of the severity of depressive symptomatology. Using a 4-choice Likert-type scale (0 = not at all; 1 = several days; 2 = more than a week; 3 = nearly every day), it evaluates the presence of 9 depressive symptoms in the last 2 weeks. A maximum score of 27 points can be obtained, and different levels of intensity can be differentiated (scores of 5–9 are classified as mild depression; 10–14 as moderate depression; 15–19 as moderately severe depression; ≥20 as severe depression). The Spanish adaptation has an excellent internal consistency (*α* = 0.89). The internal consistency for our study sample was adequate (*α* = 0.86).

*Purpose-In-Life Test-10 Part 1* (PIL-10; García-Alandete et al., 2013). This is the reduced version of the PIL (Crumbaugh and Maholick, 1969), and its main objective is to assess the level of meaning-in-life achievement experienced by individuals. It is composed of 10 items with a 7-point Likert-type response (ranging from 1 to 7, with 4 representing a neutral position), to assess different dimensions of MIL: (1) enthusiasm versus boredom, (2) excitement in life, (3) presence of clear life goals, (4) newness of each day, (5) desires for other lives, (6) activity after retirement, (7) good things in life, (8) reason for being alive, (9) ability to find meaning, and (10) presence of goals. The total score ranges from 10 to 70, with higher scores indicating greater perceived MIL. Increased internal consistency for the total PIL-10 (*α* = 0.86) is derived from the Spanish version of the instrument. In the present study, the internal consistency was slightly higher than in the original study (*α* = 0.92).

### Procedure

This investigation is part of a larger project that aims to analyze the risk and protective factors regarding the development of emotional disorders in Spanish university students. Among the variables analyzed, in addition to those included in this study, are personality traits, problem-solving skills, and perceived social support.

First, this study was approved by the ethical committee of the Catholic University of Valencia (code UCV2019-2020-087). Then, incidental sampling was performed with significant informants from different centers. Next, the deans and vice-deans of the different faculties were contacted to request authorization to pass the study protocol. After their authorization, the application of the protocol was agreed with the teaching staff. Participants were recruited in their classrooms and gave their informed consent. Participation was voluntary and they received no compensation. Data were collected from September 2020 to October 2021. Finally, an online platform was used to answer the questionnaires so that students could access them via a link or QR code. The database was correctly protected according to the Spanish data protection law.

### Data analysis

Descriptive, correlational, and then regression analyses were performed. Mediation analysis was performed using Process Macro for SPSS (Hayes, 2015) to test the hypothesis that meaning in life mediates the effect of ER strategies on depressive symptomatology in university students. Thus, regression-based mediation procedures were run employing bootstrapping techniques using 10,000 samples (Hayes, 2009; MacKinnon and Fairchild, 2009), as well as recent research (Farina et al., 2020).

We evaluated the assumptions for both the linear regression model and the mediation model. Specifically, we examined the normality assumption using the Kolmogorov test, with a significance level of *p* > 0.05, to ensure that the data followed a normal distribution. Additionally, we tested the homoscedasticity assumption using the Levene test, with a significance level of *p* < 0.05, to verify that the variability of the residuals was constant across different levels of the predictors. Furthermore, prior to conducting the model analysis, we standardized the variables to facilitate meaningful comparisons. For the mediation relationships, we employed Model 4 according to Hayes (2017) with a 95% confidence interval. To assess the significance of the variables within the model, we considered *p*-values <0.01, indicating a strong level of significance. Additionally, we examined the lower and upper limits of the confidence interval (LLCI and ULCI) to gain insights into the range of plausible values for the effects observed. To ensure robust results, we performed a bootstrapping procedure with 10,000 samples, which helps to estimate the sampling distribution of the model parameters and provides more reliable inferences.

**Figure 1:**
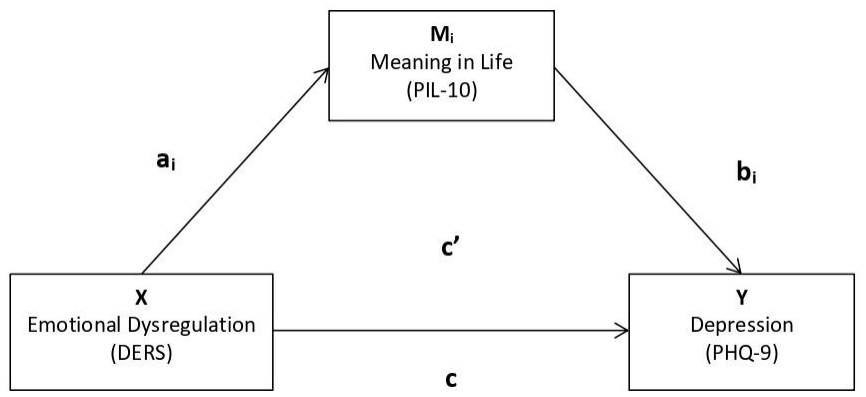
Mediation model to test and its paths.

More specifically, a regression coefficient (and associated t-test) was first calculated on the mediating variable M (and its inherent paths a and b), the independent variable X on the dependent variable without the inclusion of the moderator (path c’), and the independent variable X on the dependent variable after including the mediator (path c). Theoretical Figure 1 illustrates this analysis in terms of variables and paths. Then, the mediation analyses were performed with the DERS sub-scores, with the PIL-10 total score and PHQ-9 total score. Thus, by using Hayes’ model, we seek to test the mediating effect of MIL on the relationship between the DERS ER subfactors and depression.

## Results

To achieve the aim of this investigation, descriptive and correlational analyses were performed first, followed by the mediation model. Table 1 shows the descriptive data in terms of mean and standard deviation of the different scales. As the table shows, the assumption of normality of variables was not reached through the Kolmogorov–Smirnov test (all *p* > 0.05). Nevertheless, the distribution of the variables under study and the skewness and kurtosis were reviewed, which in no case exceeded the value of <1.50.

**Table 1:** Descriptive statistics for the DERS subfactors, total PIL-10 and PHQ-9.

	DERS					PIL-10	PHQ-9
Variable	Inattention	Confusion	Non-acceptance	Interference	Lack of control	Meaning in life	Depression
Mean	1.32	1.21	1.24	2.02	1.16	10.86	7,96
SD	0.78	0.83	0.98	1.05	0.89	2.05	5,50
Minimum	0	0	0	0	0	2.83	0
Maximum	4	4	4	4	4	14	27

*N* = 566, with no missing values.

Secondly, regarding the relationship between difficulties in ER and Depression, a positive, small and statistically significant correlation was obtained between *Depression* and *Inattention* (*r* = 0.24, *p* ≤ 0.001), whilst a positive, moderate and statistically significant correlation between *Depression* and *Confusion* (*r* = 0.46, *p* ≤ 0.001), *Non-Acceptance* (*r* = 0.57, *p* ≤ 0.001), *Interference* (*r* = 0.51, *p* ≤ 0.001), and *Lack of control* (*r* = 0.64, *p* ≤ 0.001).

Continuing with the relationship between Depression and MIL, we found a negative, moderate, and statistically significant correlation between *Depression* and *Meaning in Life* (*r* = −0.56, *p* ≤ 0.001). Moreover, the relationship between ER and MIL was analyzed. The results can be seen in Table 2. Regarding the relationship between difficulties in ER and MIL, a negative, small and statistically significant correlation has been obtained between the variable *Inattention* and *Meaning in Life* (*r* = −0.35, *p* ≤ 0.001). Likewise, for the *Confusion* variable, a small and statistically significant negative correlation was obtained with the *Meaning in Life* variable (*r* = −0.38, *p* ≤ 0.001). Continuing with the variable *Non-Acceptance*, a small and statistically significant negative correlation was obtained with the variable *Meaning in Life* (*r* = −0.36, *p* ≤ 0.001). A small and statistically significant negative correlation was also obtained between the variable *Interference* and the variable *Meaning in Life* (*r* = −0.31, *p* ≤ 0.001). The variable *Lack of control* shows a negative, small and statistically significant correlation with the variable *Meaning in Life* (*r* = −0.38, *p* ≤ 0.001).

**Table 2:** Correlation coefficients between the different emotional dysregulation strategies, meaning in life and depression.

	DERS					PIL-10	PHQ-9
	Inattention	Confusion	Non-acceptance	Interference	Lack of control	Meaning in life	Depression
Inattention	–						
Confusion	0.52***	–					
Non-Acceptance	0.23***	0.48***	–				
Interference	0.11**	0.33***	0.49***	–			
Lack of control	0.23***	0.52***	0.70***	0.65***	–		
Meaning in Life	−0.35***	−0.38***	−0.36***	−0.31***	−0.38***	–	
Depression	0.24***	0.46***	0.57***	0.51***	0.64***	−0.56***	–

**p < 0.01, ***p < 0.001.

**Figure 2:**
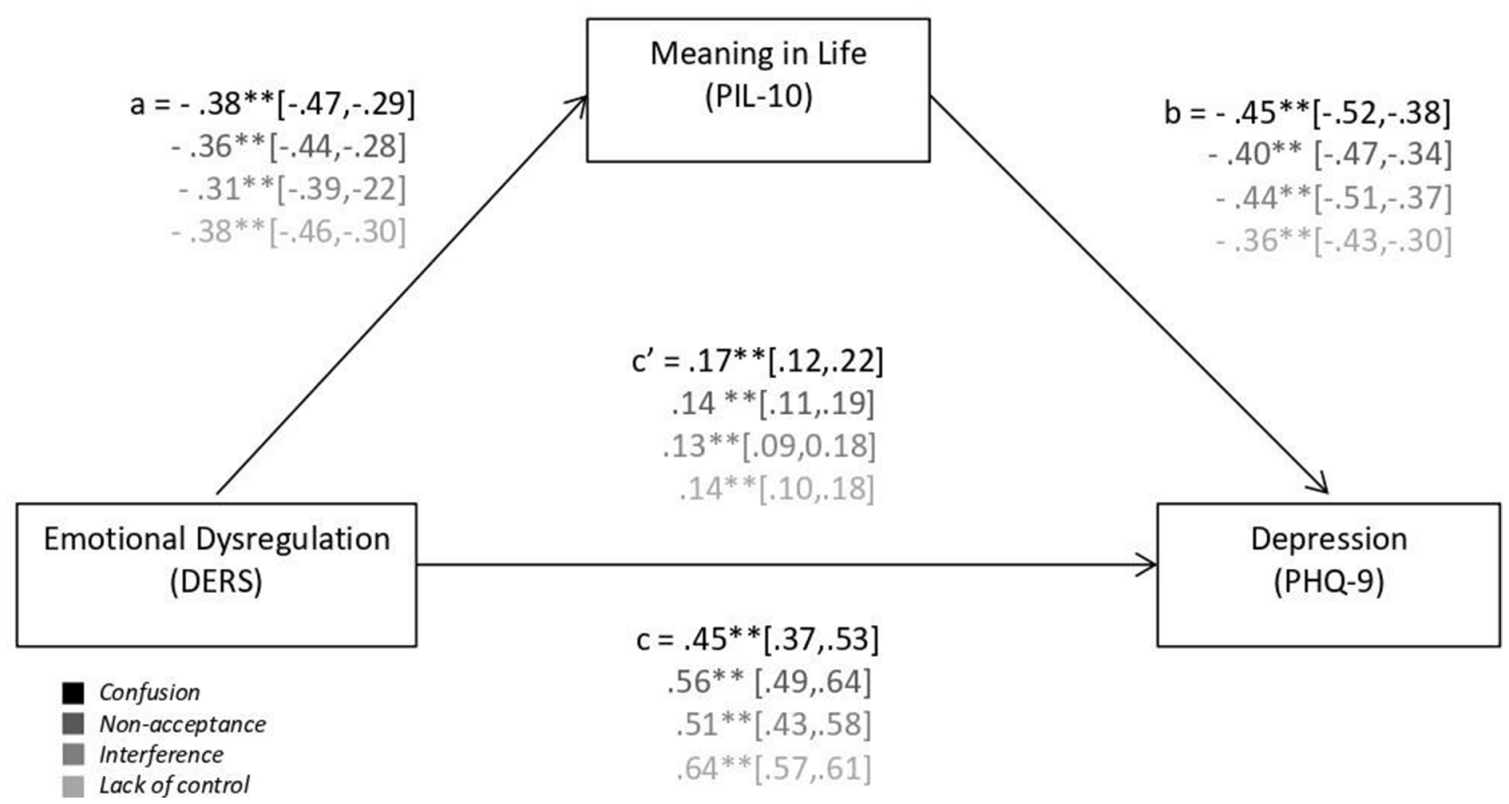
Mediation model of the meaning in life variable (PIL-10) on the emotional dysregulation (DERS) sub-factors and depression (PHQ-9). Letters a, b, and c reflect the standardized coefficient of the direct regression weight (β). c’ refers to the indirect effect. Different colors reflect the DERS subfactors. CI is reflected in square brackets. **p < 0.01.

Finally, a mediation model was tested according to the theoretical model specified in Figure 1, where MIL mediates the relationship between ER and Depression. Given that the ER scale contemplates five dimensions, the five mediation models were tested according to this approach, as shown in Figure 2.

In this way, each path outlined in the model was thoroughly examined for each model based on the different DERS subfactors. The first model of *Inattention* did not reach the level of statistical significance for the path between this variable and *Depression*, although the general model reached the level of statistical significance (*p* < 0.05). As shown in Figure 2, the *Confusion* model reached the level of statistical significance: *F*(1,566) = 73.70; MSE = 0.84; *p* < 0.001, explaining 14% of the variance. As for the *Non-Acceptance* model, it reached the level of statistical significance: *F*(1,566) = 73.004; MSE = 0.86; *p* < 0.001; explaining 13% of the variance. Continuing with the *Interference* model, it attained the level of statistical significance: *F*(1,566) = 49.67; MSE = 0.90; *p* < 0.001; explaining 9% of the variance. Finally, the *Lack of control* model reached the level of statistical significance: *F*(1,566) = 83.13; MSE = 0.85; *p* < 0.001; explaining 14% of the variance.

As illustrated in Figure 2, the various pathways, including the total effect (c) and indirect effect (c’), demonstrated statistical significance based on both the value of *p* and the confidence intervals. For this reason, the lower and upper limits for a 95% confidence interval are included within brackets. It is important to note that the confidence intervals, indicated by the brackets, did not encompass the value of zero in any instance.

## Discussion

The aim of the current study was to analyze the mediating role that MIL has between ER difficulties and depressive symptomatology in Spanish college students. First, the correlations between the different variables were analyzed to justify the approach of the mediation model, according to the literature reviewed.

On one hand, considering the results of the correlations between ER difficulties and depression, it was observed that the strategy of *Inattention* is related to depressive symptomatology. Although significant, this relationship is small. This strategy refers to the difficulty in focusing attention and devoting attentional resources to emotional information (Gratz and Roemer, 2004). This relationship could be explained because, although this strategy is important, it does not imply that after attending to emotional information, adaptive or maladaptive ER strategies are employed. Thus, even though this is an important preliminary step, it may not have as much effect on depressive symptomatology. This is aligned with other research that has studied the effects of this variable in relation to depression (Extremera et al., 2006; Hervás, 2011).

On the other hand, *Confusion*, understood as the difficulty to have an adequate awareness and understanding of emotions, showed a significant relationship with depressive symptomatology. In this sense, the difficulty in identifying emotions and understanding the causes that have generated them may prevent adequate emotion regulation (Gross, 2015; Van Beveren et al., 2018). Continuing with the *Non-Acceptance* strategy, this has also shown a significant relationship with depressive symptomatology. *Non-acceptance* is understood as the difficulty to accept emotional states which could generate a greater tendency to ruminate (Watkins and Moulds, 2005). Therefore, as the literature points out, difficulty in accepting emotions, would be related to depressive symptomatology (Schäfer et al., 2017). Finally, *Interference* and *Lack of control* have also shown a significant relationship with depression. These strategies make it difficult to engage in goal-directed behavior and increase the feeling of overwhelm due to the emotional intensity and sense of persistence of negative emotional states (Gratz and Roemer, 2004; Hervás and Jódar, 2008). Therefore, our results show that difficulties in ER are associated with depressive symptomatology. These results are in line with different studies (Radkovsky et al., 2014; Dryman and Heimberg, 2018; Visted et al., 2018; Everaert and Joormann, 2019; Reyes-Martín et al., 2022) that show that maladaptive strategies play a significant role in the onset and maintenance of depressive disorders.

Likewise, in the present study, a negative correlation between MIL and depression is revealed, in line with other studies (Psarra and Kleftaras, 2013; Volkert et al., 2014). This can be framed according to Frankl’s (2006) model which states, among other things, that human beings need to find MIL as a primary motivational factor. Furthermore, low levels of MIL would act as a risk factor against the development of emotional disorders, while higher levels of MIL would act as a protective factor against psychopathology. Also, longitudinal studies have confirmed the predictive and moderating effect of MIL against depression (Disabato et al., 2017). Therefore, there is a broad consensus among researchers on the relationship between low levels of MIL and depressive symptoms (Marco et al., 2016; Sun et al., 2022).

Also, we found a negative correlation between emotional dysregulation and MIL, suggesting that MIL is a factor that significantly influences ER processes. This result suggests that the use of adaptive or maladaptive ER strategies could hinder or favor the creation of MIL and vice versa (Marco et al., 2017a, 2020b, 2021). Different studies have found that in the presence of high MIL, better ER (Park and Yoo, 2016), better emotion-focused coping and positive affect are observed (Ward et al., 2023), confirming the important relationship between both constructs (Abeyta et al., 2015; Marco et al., 2017b; García-Alandete et al., 2018).

Moreover, the results of the mediation model confirmed that MIL is a mediating factor between emotional dysregulation and depressive symptomatology. This result suggest that low levels of MIL may increase negative emotions and emotional instability, increasing depressive symptomatology, hindering an adequate use of adaptive ER strategies and interfering or hindering the creation of sense of MIL (Marco et al., 2020a, 2021). On the other hand, MIL could attenuate the impact of maladaptive ER strategies on depressive symptomatology (Marco et al., 2017a,b, 2020a). These results are supported by other studies that point to the importance of MIL as a mediating variable in different variables related to depressive symptomatology, among which hopelessness and emotional dysregulation stand out, both in a clinical population with borderline personality disorder and suicide risk (Marco et al., 2014; Lew et al., 2020) and in non-clinical adolescent population (García-Alandete et al., 2018).

In our mediation model, it is observed that all the emotional dysregulation subfactors (*Emotional Confusion, Non-Acceptance, Emotional Interference, and Lack of emotional control*), except *Emotional Inattention*, are similar and perform well in the model meaning that they influence in the development of depressive symptomatology, mediated by the MIL. In this sense, several investigations also show that people with higher levels of depressive symptomatology present greater attention to emotions, high levels of *emotional confusion*, greater affective dysregulation and greater *Non-acceptance* (Ehring et al., 2008 Hervás, 2011). This means that people with depressive symptomatology present difficulties in analyzing and emotionally processing events of negative valence (Greenberg and Watson, 2006; Liu and Thompson, 2017). *Emotional Inattention* is the only factor that does not perform well in the model. This result may be due to the fact that, as Hervás and Jódar (2008) state, attention to emotions can be functional or dysfunctional depending on the individual’s regulatory capacity (Lischetzke and Eid, 2003), since the rest of the subscales (*Emotional Confusion, Non-Acceptance, Emotional Interference and Emotional Lack of control*) represent content that is unequivocally maladaptive.

Finally, it could be noted that high levels of MIL could act as a protective factor against depression as it contributes to experiencing a higher quality of life, well-being, and better health and psychological functioning (Hedberg et al., 2011; Heintzelman and King, 2014; Krok, 2017; Yang and Wu, 2021). It can also buffer the effect of stressful interpersonal events by redirecting emotional response and supporting better recovery and more adaptive coping (Park and Baumeister, 2017; Ward et al., 2023).

### Limitations and implications

The current study has several limitations that should be considered. First, it is a survey study, so the scores obtained are self-reported, and there may be biases in the participants’ responses. For future lines of research, it would be interesting to explore the integration of additional evaluation measures, such as the Ecological Momentary Assessment (EMA) (Shiffman et al., 2008). Incorporating EMA would enable the conduct of longitudinal studies and enhance the ecological validity of the results. Furthermore, this is a cross-sectional study, which implies that a causal relationship cannot be established between the variables analyzed. In future research, it would be interesting to carry out a longitudinal study.

At a theoretical level, this study is novel because it addresses the mediating role of MIL between ER and depressive symptomatology. Therefore, it expands the information on the implication on these variables and their interrelation in models of psychopathology. From the applied point of view, this study allows the development of prevention and/or treatment programs that promote an adequate MIL, in addition to incorporating this variable in existing multi-component programs that include ER. Among them, the Unified Protocol for Transdiagnostic Treatment of Emotional Disorders (Barlow et al., 2011a,b), which draw attention to ER, could be mentioned suggesting that adding MIL to them could be beneficial. In addition, this study highlights the usefulness of other multicomponent intervention programs such as Dialectical Behavior Therapy (DBT) (Linehan, 1993) or Acceptance and Commitment Therapy (ACT) (Hayes and Lillis, 2012) that include MIL as an important component of therapy work (Hill, 2018).

## Data availability statement

The raw data supporting the conclusions of this article will be made available by the authors, without undue reservation.

## Ethics statement

The studies involving human participants were reviewed and approved by Universidad Católica de Valencia Institutional Review Board (Code #: UCV2019-2020-087). The patients/participants provided their written informed consent to participate in this study.

## Author contributions

All authors listed have made a substantial, direct, and intellectual contribution to the work and approved it for publication.

## Funding

This work was supported by Universidad Católica San Vicente Mártir; Under grant UCV 2023-255-002.

## Conflict of interest

The authors declare that the research was conducted in the absence of any commercial or financial relationships that could be construed as a potential conflict of interest.

## Publisher’s note

All claims expressed in this article are solely those of the authors and do not necessarily represent those of their affiliated organizations, or those of the publisher, the editors and the reviewers. Any product that may be evaluated in this article, or claim that may be made by its manufacturer, is not guaranteed or endorsed by the publisher.
